# Development of biodegradable polycaprolactone film as an internal fixation material to enhance tendon repair: an *in vitro* study

**DOI:** 10.1186/1471-2474-14-246

**Published:** 2013-08-19

**Authors:** Jian-Zhong Hu, Yong-Chun Zhou, Li-Hua Huang, Hong-Bin Lu

**Affiliations:** 1 Department of Spine Surgery, Xiangya Hospital, Central South University, Changsha 410008, China; 2 Center for Medical Experiments, Third Xiangya Hospital, Central South University, Changsha 410013, China; 3 Department of Sports Medicine, Research Center of Sports Medicine, Xiangya Hospital, Central South University, Changsha 410008, China

**Keywords:** Tendon injury, Internal fixation, Biomechanics, Microstructure

## Abstract

**Background:**

Current tendon repair techniques do not provide sufficient tensile strength at the repair site, and thus early active motion rehabilitation after tendon repair is discouraged. To enhance the post-operative tensile strength, we proposed and tested an internal fixation technique using a polycaprolactone (PCL) biofilm. PCL was chosen for its good biocompatibility, excellent mechanical strength, and an appropriate degradation time scale.

**Methods:**

PCL biofilms were prepared by a modified melt-molding/leaching technique, and the physical and mechanical properties and *in vitro* degradation rate were assessed. The pore size distribution of the biofilm and the paratenon of native tendons were observed using scanning electron microscopy. Next, we determined whether this biofilm could enhance the tensile strength of repaired tendons. We performed tensile tests on rabbit Achilles tendons that were first lacerated and then repaired: 1) using modified Kessler suture combined with running peripheral suture (‘control’ group), or 2) using biofilm to wrap the tendon and then fixation with sutures (‘biofilm’ group). The influence of different repair techniques on tendon tensile strength was evaluated by mechanical testing.

**Results:**

The novel biofilm had supple texture and a smooth surface. The mean thickness of the biofilm was 0.25 mm. The mean porosity of the biofilm was 45.3%. The paratenon of the rabbit Achilles tendon had pores with diameters ranging from 1 to 9 μm, which were similar to the 4–12 μm diameter pores in the biofilm cross-section. The weight loss of the biofilms at 4 weeks was only 0.07%. The molecular weight of PCL biofilms did not change after immersion in phosphate buffered saline for 4 weeks. The failure loads of the biofilm were similar before (48 ± 9 N) and after immersion (47 ± 7 N, *P* > 0.1). The biofilm group had ~70% higher mean failure loads and 93% higher stiffness compared with the control group.

**Conclusions:**

We proposed and tested an internal fixation technique using a PCL biofilm to enhance tendon repair. Internal fixation with the biofilm followed by standard suturing can significantly increase the tensile strength of tendon repair sites. This technique has the potential to allow active motion rehabilitation during the early post-operative period.

## Background

Tendon injuries are common and surgical repair is often necessary. During the healing process, the most common complication is adhesion formation, which restricts tendon gliding. Historically, repaired flexor tendons were immobilized (fixed in place with an external brace during the first few post-operative weeks) until sufficient healing had occurred to allow motion of the tendon [1]. Thus, adhesion formation inevitably occurred and led to finger dysfunction. During surgical repair, avoidance of tendon trauma and preservation of the anatomic integrity of normally contiguous structures may decrease the adhesion formation [2,3]. Since about the 1970s, methods of mobilizing repaired tendons have been developed allowing limited passive motion to promote gliding of the tendon during the post-operative period [4-6]. Early passive mobilization might improve tendon healing and enhance tendon gliding and tensile strength at the repair site [7-11], but may also cause the tendon to buckle, roll, or fold up [12]. Over the last three decades, controlled active motion immediately after repair was proposed and tested [13,14]. Controlled active motion protocols with conventional suture can produce a high percentage of excellent and good outcomes [15,16]. However, because of the difficulties in controlling the amount of load across the repair site during active motion rehabilitation, this technique is also associated with a high rupture rate [17-19].

The optimal tendon repair technique would not only provide coaptation without gap formation at the repair site, but would also provide sufficient strength to safely allow active mobilization to reduce adhesion formation [20]. Immediate active mobilization of repaired tendons was thought to be the most effective way to restore function, and could decrease or even eliminate adhesions [21-24]. However, in the early period after surgery, the strength of the repair depended completely on the repair materials and suture techniques.

Currently, popular techniques for tendon repair (e.g. the modified Kessler suture plus running peripheral suture) [25-29] do not provide sufficient tensile strength at the repair site to recommend early active mobilization. We hypothesized that wrapping a polycaprolactone (PCL) biofilm around the repair site and fixing it in place would increase the tensile strength of the repaired tendon, creating a stable repair site for post-operative early active mobilization.

PCL was chosen as the material for the internal fixation biofilm because PCL is a non-toxic biodegradable polyester, has a slow degradation rate, and demonstrates excellent mechanical strength and biocompatibility [30-36]. In this study, we examined the material properties of the PCL biofilm to determine whether it could be used to strengthen tendon repairs.

## Methods

### Experimental design

Biofilms were prepared using PCL, and the thickness, porosity, pore size, and *in vitro* degradation rate were measured. Three aspects of biofilm degradation were investigated: the weight, the molecular weight, and the failure load after 4 weeks in solution.

**Figure 1:**
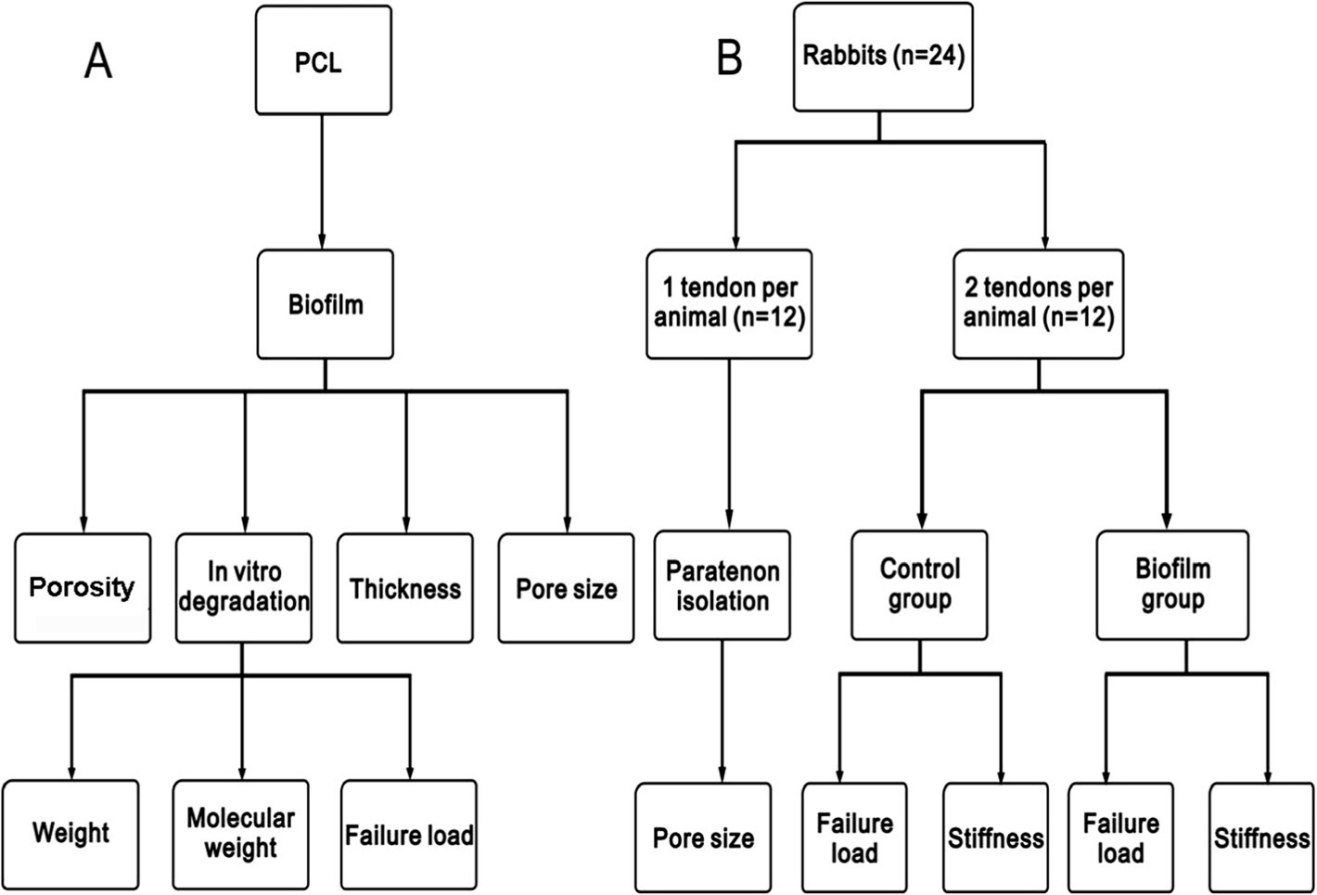
**A flowchart of the experimental design. (A)** Biofilm specimen preparation and *in vitro* evaluation. **(B)** Animals used, tendons harvested, experimental groups, and outcome measures.

To assess the ability of the PCL biofilm to enhance the tensile strength of repaired tendons, we used a paired group design for tensile testing of repaired rabbit Achilles tendons. One group was repaired using standard repair techniques (‘control’ group), while the second group was repaired using the PCL biofilm to wrap and fix the repair site (‘biofilm’ group). Twelve animals in total were used, with two operated digits per animal – one each for the control and biofilm groups. In a second group of 12 animals, the paratenon of one tendon per animal (12 tendons in total) were stripped and used for observation of the pore size distribution of the tendon outer membrane. A flowchart summarizing the experimental design is shown in Figure 1. The animal protocol used in the current study was approved by the ethics review committee of the Third Xiangya Hospital, Central South University.

### Biofilm specimen preparation

All chemicals were purchased from Alfa Aesar unless otherwise specified. PCL (molecular weight: 200,000 Da) was purchased from Yisheng New Materials Co., Ltd., Shenzhen, China. Before use, tetrahydrofuran (THF) and *N,N*-dimethylformamide (DMF) were purified to remove water. Briefly, THF was put in calcium hydride for reflux for 12 h and redistilled, and DMF was stirred in calcium hydride (5% w/v) for 12 h, filtered and then distilled at 20 mmHg. A modified melt-molding/leaching technique was used to prepare the biofilms, using DMF as the porogen. The blending of PCL, THF, and DMF was carried out in a beaker on a magnetic stirrer (HPC07SA-P, APLUS, Union city, CA, USA) operating at 45°C and a stirring speed of 200 rpm. PCL (2 g) was added to 20 ml of THF and mixed for 15 min to prepare a homogeneous solution. Next, 1.7 ml of DMF was added and the solution mixed for another 5 min. The resulting blended solution was placed into a rectangular mold of dimension 140 × 100 × 10 mm, and dried at 25°C until the THF had completely evaporated. To obtain the biofilm, the shaped PCL-DMF blends were immersed in a continuous shaking deionized water bath at 37°C to leach out the DMF. Fresh changes of deionized water were performed every 12 h until the wet specimen reached a constant weight, confirming that all the DMF was removed. Finally, the specimen was lyophilized for 24 h and stored in a dry container until use.

### Determination of the porosity and thickness of the biofilm

The porosity of the specimen was measured by Archimedes’ method. First, the specimen was lyophilized to determine the dry weight (W_1_). Then, the specimen was transferred to a beaker and placed in a vacuum oven at 2 kPa for 15 min, followed by slow injection with water until the specimen was completely immersed. Next, the pressure was gradually restored to atmospheric, and then the saturated specimen was placed in a copper wire basket, suspended in a beaker filled with water, and weighed (W_2_). The specimen was taken out of the water, any water remaining on its surface was removed with wet gauze, and the specimen was weighed again (W_3_). The porosity was calculated using the following formula: porosity (%) = [(W_3_ – W_1_)/(W_3_ – W_2_)] × 100% (n = 12). Thickness was measured using a micrometer (n = 12).

### *In vitro* degradation of the biofilm

The biofilm was cut into 100 mm × 20 mm pieces for *in vitro* degradation testing. Lyophilized specimens were accurately weighed (W_0_), and then immersed in phosphate buffered saline (PBS) pH 7.4 in a continuous shaking water bath at 37°C for 4 weeks. The degradation medium of PBS was changed each week. After 4 weeks, the specimens were removed, washed with deionized water, lyophilized, and reweighed (W_F_). The percent weight loss was calculated as: weight loss % = [(W_0_ − W_F_)/W_0_] × 100% (n = 12). After 4 weeks of degradation, PCL biofilms were dissolved in THF at a concentration of 1 mg/mL and filtered through a 0.2-μm inorganic membrane filter (GE Healthcare, UK). The molecular weight of the biofilm was determined by gel permeation chromatography (GPC apparatus 1525, Waters Co., Milford, MA, USA) (n = 12).

In order to examine the tensile strength, the specimens were fixed in custom-made clamps and mounted in a microcomputer-controlled electronic universal mechanical testing machine (Dual Column Testing System 3369, Instron Co., Norwood, MA, USA). Before testing, both the ends (10 mm) of the specimen were first wrapped with tape and then clipped in the clamps equipped with rubber pads. The initial distance between the fixtures was set at 80 mm. The specimens were distracted at a rate of 5 mm/min until failure. During the testing, if the specimen slipped away from the clamps, or fracture occurred within 10 mm of any clamp, the measurement was considered invalid. Throughout the testing process, fracture occurred in the middle of all specimens and no specimen slipped away from the clamps.

### Microstructural characterization of the biofilm and the Achilles tendon outer membrane

Twelve rabbits were euthanized and 1 Achilles tendon was harvested from each. The peeled paratenon of the Achilles tendons were fixed with 2.5% glutaraldehyde, dehydrated by alcohol gradients, soaked in osmium tetroxide, dried and coated with gold, and then the pore size distribution of the tendon outer membrane was observed with field emission scanning electron microscopy (FESEM, Nova NanoSEM 230, FEI Co., Hillsboro, OR, USA) (n = 12). The prepared biofilms were placed in liquid nitrogen for 30 min, broken to pieces, freeze-dried, and evenly sprayed with gold. Finally, the cross-section was observed with FESEM (n = 12).

### Establishment of an *in vitro* model of Achilles tendon repair

**Figure 2:**
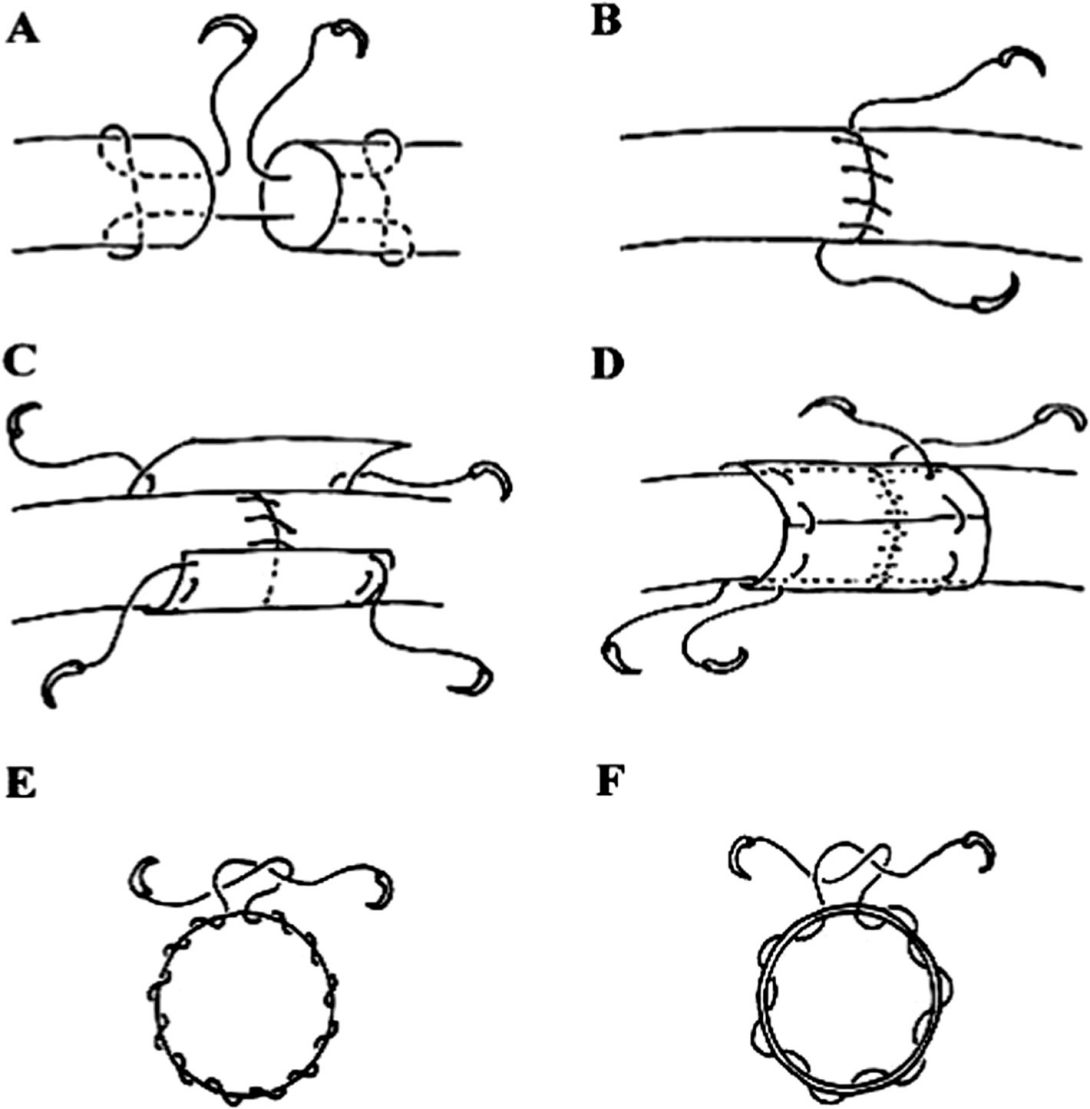
**Schematic of tendon repair techniques. (A)** Modified Kessler suture: 4–0 polyester suture placed in the tendon and tied with a single buried knot with three throws. **(B)** Running peripheral suture: 6–0 polyester suture placed in a running fashion. **(C)** Biofilm-wrapped Achilles tendon: after repairing with standard techniques, the Achilles tendon was wrapped with biofilm, with 5 mm lengths of biofilm on both sides of the lacerated site. **(D)** Biofilm fixation: 6–0 polyester suture placed in a running fashion. **(E)** Cross-section of a laceration repaired by the running superficial peripheral suture technique. Here, the needle passage was through the paratenon and only the superficial tendon. **(F)** Cross-section of a suture site for biofilm fixation after wrapping: using the running superficial suture technique, the biofilm was fixed on the Achilles tendon. The needle passage was through the biofilm, the paratenon, and only the superficial tendon.

Twelve more rabbits were euthanized and 24 Achilles tendons were harvested, two from each animal (length = 4 cm). The Achilles tendons were sharply cut into two parts (2 cm long each). For the control group, each tendon repair was conducted using the modified Kessler method (Figure 2A) with 4–0 polyester suture (DemeTECH, Miami, FL, USA) and the running peripheral suture (Figure 2B) with 6–0 polyester suture (DemeTECH). For the biofilm group, the tendons were repaired with standard repair techniques, wrapped with PCL biofilm (Figure 2C), and then sutured with 6–0 polyester suture (Figure 2D). For the running superficial peripheral suture, the needle passage was through the paratenon and only the superficial tendon (Figure 2E). For fixation of the biofilm, the needle passage was through the biofilm, the paratenon, and only the superficial tendon (Figure 2F).

### Biomechanical testing of the tendon repair model

The repaired tendons were fixed in the universal mechanical testing machine clamps and distracted at a rate of 0.3 mm/min (n = 12). The length of tendon between the clamps was kept constant at 3 cm. The load–displacement curves were simultaneously traced by the testing machine computer. The failure load was defined as the peak load of the repair construct. The stiffness of the repair was calculated as the slope of the linear region of the load–displacement curve. The entire testing process was conducted at a room temperature of 25°C with 70% relative humidity, and the specimens were kept moist with saline spray.

### Statistical analysis

All data obtained are presented as mean ± SD. One-way ANOVA with paired t-tests were performed to detect significant (*P* < 0.05) effects of the experimental variables.

## Results

### The biofilm had excellent physical properties

**Figure 3:**
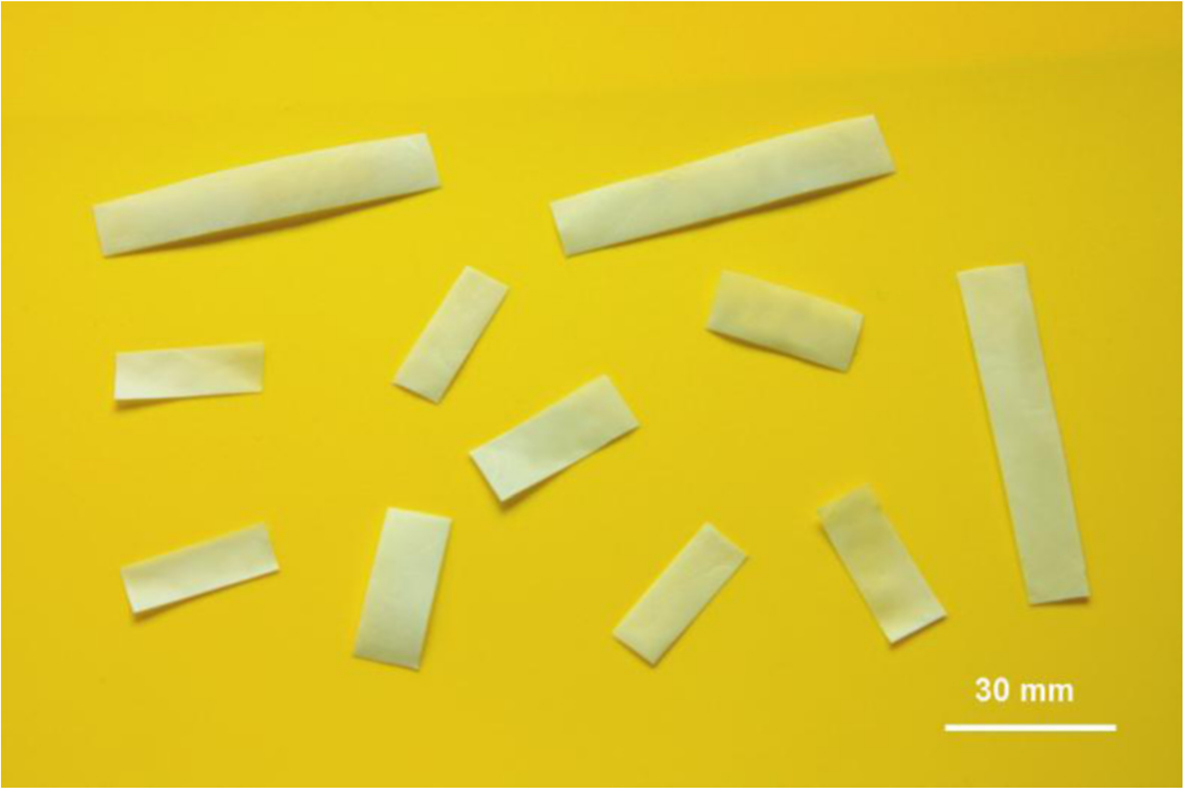
**Image of biodegradable PCL film.** The biofilm had a supple texture and smooth surface, and was surgically manageable and easily incorporated at the repair site.

The thickness of the biofilm was 0.25 ± 0.06 mm, and the porosity was 45.3 ± 5%. The biofilm had supple texture and smooth surface (Figure 3), and was surgically manageable and easily incorporated at the repair site.

### The biofilm degraded slowly *in vitro*

The biofilm degradation rates and the influence of early degradation on the mechanical properties of the biofilm were observed *in vitro*. All specimens showed very slow degradation rates after soaking for 4 weeks in PBS. The weight loss of the biofilms was only 0.07 ± 0.005% after 4 weeks.

The molecular weight of the biofilm showed no change from 200,000 Da after 4 weeks soaking in PBS (data not shown). The failure loads of the biofilm were similar before (48 ± 9 N) and after 4 weeks of degradation (47 ± 7 N, *P* > 0.1). These results were correlated with the minimal weight loss.

### The pore size distribution of the PCL biofilm was similar to that of the Achilles tendon paratenon

**Figure 4:**
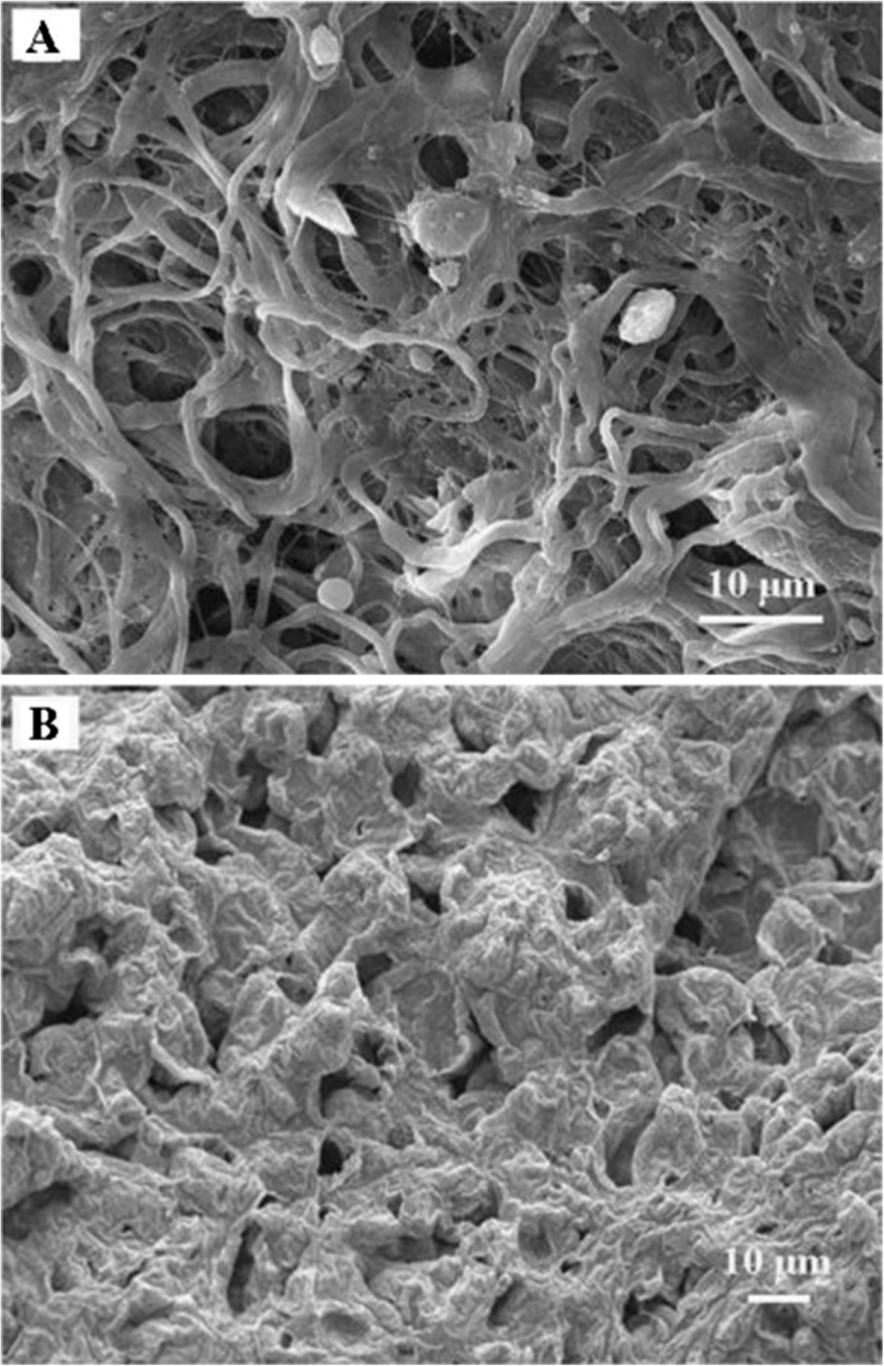
**FESEM images of the tendon outer membrane and biofilm cross-section. (A)** The pores of rabbit Achilles tendon outer membrane had diameters from 1 to 9 μm. **(B)** The pores of the cross-section of the biofilm had diameters from 4 to 12 μm. Three randomly chosen fields of view were photographed from each specimen. Representative images are shown.

FESEM was used to examine the pore size distribution of the tendon outer membrane and the biofilm. Representative FESEM images of the biofilm and the tendon outer membrane display similar pore diameters (Figure 4). An examination of twelve specimens (3 random images for each specimen) showed that the pore size of the rabbit Achilles tendon outer membrane had diameters ranging from 1 to 9 μm. The pore size of the cross-section of the biofilm had diameters ranging from 4 to 12 μm.

### Internal fixation with the biofilm can significantly increase tensile strength of the tendon repair site

The mean failure load of the control group was 23.5 ± 4.9 N, while that of the biofilm group was 39.6 ± 6.7 N (*P* < 0.05), an increase of almost 70%. The mean stiffness of the control group was 7.11 ± 1.9 N/mm, while that of the biofilm group was 13.69 ± 5.8 N/mm (*P* < 0.05), an increase of almost 93%.

## Discussion

Many different repair methods have been explored with the aim of enhancing the tensile strength of repaired tendons [37-40]. For various reasons, however, these methods are not widely used in clinics. For example, Becker et al. [37] reported a technique involving beveling of the tendon ends and fine compressive suturing. The drawback of this approach is that it causes shortening of the tendon ends. The double loop locking suture technique reported by Lee [38] and the fish mouth anastomosis method described by Pulvertaft [39] were able to strengthen the repair; however, normal tendon anatomy is distorted with these methods. The cross-stitch plus mesh sleeve technique described by Silfverskiöld [40] is time-consuming and technically complicated.

Currently, the modified Kessler suture plus running peripheral suture is the most popular tendon repair technique [28]. However, this suture technique cannot develop enough strength at the suture site, and early active motion may result in a higher incidence of rupture. Therefore, new materials and techniques for increasing the strength of tendon repair sites are needed for hand surgery.

This study presented a biofilm for internal fixation that increased the failure load of *in vitro* tendon repair by 70% over standard techniques, and, therefore, might allow for the initiation of early active motion rehabilitation.

PCL was chosen because of its desirable material properties, such as good biocompatibility and complete degradation, absorption, and excretion in the body [32,35,36,41,42]. In a previous study, rat mesenchymal stem cells were able to adhere and proliferate on PCL scaffolds for bone tissue engineering, demonstrating good biocompatibility [32]. In a different study where tritium-labeled PCL (*M*_w_: 3,000 Da) was subcutaneously implanted in rats, 92% of the implanted radioactive tracer was excreted from feces and urine by 135 days after implantation [30]. Finally, another study showed that three-dimensional PCL scaffolds (*M*_n_ 80,000) had no molecular weight change after 6 months *in vitro* and *in vivo*, revealing excellent long-term biocompatibility and no adverse host tissue reactions [35]. Very little *in vitro* degradation of the PCL film prepared (*M*_w_ 200,000) in the current study was observed after 4 weeks. Therefore, the biofilm would not lose its mechanical properties and would be able to support the injured tendon repair during the early stage of tendon healing. In addition, the high porosity of the biofilms and the size of the pores were similar to those of native tendons, suggesting that the biofilm should not impede tendon nourishment.

While this study indicated that internal fixation with PCL might enhance the strength of the repair after tendon injury and repair, the present research was only an *in vitro* investigation. Additional *in vivo* factors involved in tendon healing could be investigated, such as potentially inhibitory effects of wrapping a biofilm around the repair site on the migration of immune cells and fibroblasts to the site. Neutrophils and monocytes infiltrate the repair during the first 3 post-operative days. They play an important role in the clearance of bacteria and debris and are vital for chemotaxis of fibroblasts to the repair site [43-45]. Fibroblasts are the cell type that will eventually rebuild the tendon via the production of collagen [43-45]. Many studies have shown that immune cells and fibroblasts enter the repair site via the surface of the tendon [43-47]. While the pore size of the PCL biofilm is large enough for nutrients and cytokines to infiltrate, it appears to be too small for many of these cell types to gain access to the repair site. Neutrophils are generally 12–15 μm, monocytes are 10–30 μm, and tendon fibroblasts are 20–70 μm in length and 8–20 μm wide. Given that the pores of the biofilm are only 4–12 μm, it appears that the biofilm would inhibit cell infiltration, and thus impede tendon healing. However, many methods could be adopted to enlarge the pore size, such as increasing the DMF concentration while blending the PCL, THF, and DMF. It is also feasible to incorporate sacrificial beads into the biofilms that will degrade, leaving an increased porosity (e.g. use of salt particulate (NaCl) and water-soluble polymer (PEG) as co-porogens [36]). Further studies should focus on improving the pore size and the porosity of the biofilm, as well as its biocompatibility, degradation, absorption, excretion, and mechanical properties using an *in vivo* rabbit Achilles tendon repair model.

## Conclusions

In this study, we proposed the use of an internal fixation technique and novel material to enhance tendon repair. The biofilms used had high porosity and slow degradation rates *in vitro*. The pore size distribution of the biofilm was similar to that of the paratenon of rabbit Achilles tendon. Internal fixation with the biofilm followed by standard suture techniques significantly increased the tensile strength of the tendon repair site, and thus might allow early active motion rehabilitation without the risk of rupture. However, *in vivo* studies are needed to further explore whether the biofilms will enhance the healing properties of repaired tendons.

## Competing interests

The authors have no competing interests to declare.

## Authors’ contributions

YZ conceived the project and revised the manuscript. JH carried out the model building, biofilm specimen preparation, and drafted the manuscript. HL performed the biomechanical testing of the Achilles tendon and the biofilm, and revised the manuscript. LH carried out the microstructure characterization of the Achilles tendon and biofilm, and helped to revise the manuscript. All authors read and approved the final paper.

## Pre-publication history

The pre-publication history for this paper can be accessed here:

http://www.biomedcentral.com/1471-2474/14/246/prepub
